# Using low-moisture molasses-based blocks to supplement Ca salts of soybean oil to forage-fed beef cows

**DOI:** 10.1093/tas/txaa061

**Published:** 2020-05-15

**Authors:** Alice Poggi Brandão, Reinaldo F Cooke, Kelsey M Schubach, Eduardo A Colombo, Giovanna N Scatolin, Bruna Rett, Donald B Jump, Ky G Pohler

**Affiliations:** 1 Department of Animal Science, Texas A&M University, College Station, TX; 2 Nutrition Program, School of Biological and Population Health Sciences, Linus Pauling Institute, Oregon State University, Corvallis, OR

**Keywords:** beef cows, Ca salts of soybean oil, fatty acids, low-moisture block, supplementation

## Abstract

This experiment compared plasma fatty acid (FA) profile of forage-fed beef cows receiving a molasses-based supplement enriched with Ca salts of soybean oil [CSSO; 24.7% of dry matter (DM)] via a self-fed low-moisture block (LMB) or hand-fed granular concentrate daily (CONC). Thirty-six nonlactating, nonpregnant, multiparous beef cows were blocked by age (three blocks), ranked within blocks by body weight (BW) and body condition score (BCS), and allocated to 1 of three drylot pens (27 × 10 m) per block. Nine pens with four cows each were enrolled in a replicated 3 × 2 Latin square design with two periods of 42 d, and a 21-d washout interval. On day 0, pens within each block were randomly assigned to receive one of the three treatments, in a manner that pens did not receive the same treatment in both periods (total *n* = 6 pens per treatment). Cows received hay (*Cynodon dactylon*), water, and a mineral–vitamin mix for ad libitum consumption during the study. Hay intake was recorded daily from days 0 to 42, and LMB intake was recorded from days 14 to 42 to allow cows to adapt to supplement with minimal interference from days 0 to 13. The CONC was offered at 0.420 kg/cow daily (DM basis) from days 0 to 13 and then adjusted (days 14 to 42) to match LMB intake. Cow BW and BCS were recorded, and blood samples were collected on days 0, 14, 28, and 42. Average LMB intake during the initial 13 d was 0.846 ± 0.107 kg/cow daily (DM basis). Supplement DM intake did not differ (*P* = 0.39) between LMB and CONC cows from days 14 to 42 as designed (0.570 vs. 0.583 kg/d, respectively; SEM = 0.011), despite a greater variation in daily intake of LMB vs. CONC (treatment × day interaction; *P* < 0.01). No treatments effects were noted (*P* ≥ 0.40) for hay intake, BCS, and BW. Treatment × day interactions were detected (*P* ≤ 0.01) for plasma concentrations of ω-6 polyunsaturated FA and total FA. On day 0, plasma FA profile did not differ (*P* ≥ 0.20) between treatments. From days 14 to 42, plasma concentrations of linoleic acid, ω-6 polyunsaturated FA, and total FA were greater (*P* < 0.01) in CONC and LMB vs. NOSUPP cows. Plasma concentrations of these FA were also greater (*P* ≤ 0.03) in LMB vs. CONC cows on day 14, but did not differ (*P* ≥ 0.35) on days 28 and 42. These results indicate that CSSO inclusion into LMB resulted in similar incorporation of ω-6 polyunsaturated and total FA in the circulation compared with CONC offered at the same daily rate. Hence, the use of self-fed LMB appears to be a valid strategy to provide CSSO to forage-fed beef cattle with reduced labor needs.

## INTRODUCTION

Supplementing Ca salts of soybean oil (CSSO) to beef cows has been associated with productive and reproductive benefits in cow–calf systems (Cooke, 2019). For example, CSSO supplementation to beef females during the breeding season increased incorporation of ω-6 polyunsaturated fatty acid (FA) into maternal and embryonic tissues and enhanced mechanisms related to early maternal recognition of pregnancy, leading to increased pregnancy rates (Cooke et al., 2014; Cipriano et al., 2016; Brandão et al., 2018). Supplementing CSSO to beef cows during gestation has also stimulated programming effects on postnatal offspring growth and carcass quality, improving feedlot average daily gain and carcass marbling (Marques et al., 2017). Across these experiments, CSSO was mixed with granular feed ingredients (e.g., corn) and hand-fed to cows. Hand-fed supplementation demands intensive labor and increase production costs in pasture-based systems (Miller et al., 2001), which may discourage the use of CSSO supplementation by commercial cow–calf producers.

One strategy to alleviate labor demands is with the use of low-moisture molasses-based block (LMB), a self-fed form of supplementation to provide energy, protein, and custom nutrients to forage-fed cattle (Moriel et al., 2019). However, self-fed supplements such as LMB have increased intake variation compared with hand-fed granular supplements (Bowman and Sowell, 1997), which may affect duodenal absorption of CSSO and accumulation of ω-6 polyunsaturated FA in the circulation (Cooke et al., 2014). The manufacturing process of LMB includes extreme heat and changes in pH, which can also decrease ruminal stability and integrity of CSSO reaching the intestine (Sukhija and Palmquist, 1990). Hence, research is warranted to determine whether inclusion of CSSO into LMB will deliver equivalent amounts of ω-6 polyunsaturated and total FA to forage-fed beef cows compared with hand-fed granular supplements. Based on this rationale, the hypothesis of this experiment is that cows receiving CSSO via LMB will have similar plasma concentrations of ω-6 polyunsaturated FA compared with cohorts receiving CSSO daily via a hand-fed granular supplement. This experiment compared feed intake, changes in body weight (BW) and body condition score (BCS), and plasma FA profile in beef cows receiving no supplementation, or CSSO via LMB or a hand-fed granular supplement.

## MATERIALS AND METHODS

This experiment was conducted from April to July 2019 at the Texas A&M—Beef Cattle Systems (College Station, TX). All animals were cared for in accordance with acceptable practices and experimental protocols reviewed and approved by the Texas A&M - Institute of Animal Care and Use Committee (#2018-0504).

### Animals and Treatments

Thirty-six nonlactating, nonpregnant, multiparous beef cows (average 3/4 *Bos taurus* and 1/4 *Bos indicus*; initial BW = 445 ± 9 kg; initial BCS = 5.3 ± 0.06; age = 4.9 ± 0.3 yr) were assigned to this experiment. Cows were blocked by age (block A = 3.1 ± 0.1 yr; block B = 5.1 ± 0.2 yr; block C = 7.0 ± 0.1 yr). Within each block (*n* = 12 per block), cows were ranked by BW and BCS and allocated to one of three drylot pens (27 × 10 m, with 6 m of linear bunk space), in a manner that pens had similar initial average BW and BCS. Therefore, nine pens with four cows each were enrolled in this experiment, whereas cow age was used as block factor as dominant older cows may limit the access of younger cows to the LMB (Bowman and Sowell, 1997; Cockwill et al., 2000).

Pens were enrolled in a replicated 3 × 2 Latin square design containing two periods of 42 d, and a 21-d washout interval between periods. At the beginning of each period (day 0), pens within each block were randomly assigned to receive one of three treatments: 1) self-fed LMB supplement enriched with CSSO (Essentiom; Church and Dwight Co., Inc., Princeton, NJ; *n* = 6), 2) hand-fed granular supplement enriched with CSSO (Essentiom; Church and Dwight Co., Inc.) offered daily (CONC; *n* = 6), or 3) no supplementation (NOSUPP; *n* = 6). The LMB (Midcontinent Livestock Supplements Inc., Valley Mills, TX) was designed to yield a daily intake of 0.454 kg/cow (as-fed basis), and subsequent CSSO daily intake of 100 g/cow as in Brandão et al. (2018). The CONC was designed to have the same composition of the LMB, but mixed and fed daily using individual granular ingredients. Pens were not assigned to the same treatment in both periods, whereas cows were maintained as a single group in 1-hectare paddock during the washout interval. Cows received hay (*Cynodon dactylon*), water, and a mineral–vitamin mix for ad libitum consumption during both periods and the washout interval. Composition and nutritional profile of all feed ingredients and treatments are described in Tables 1 and 2.

**Table 1.** Nutritional and fatty acid profile (dry matter basis) of feedstuffs^1^

Item	Cottonseed meal	Essentiom^2^	Dry molasses	Hay
Dry matter, %	89.9	95.0	91.6	74.5
Total digestible nutrients, %	68	190	77	59
Net energy for maintenance, Mcal/kg	1.58	4.86	1.87	1.23
Crude protein, %	45.3	0.70	9.50	17.5
Neutral detergent fiber, %	25.2	1.10	1.22	49.9
Fatty acids,^3^ %	5.00	82.0	0.62	2.22
Palmitic (16:0), %	1.28	25.7	0.12	0.46
Stearic (18:0), %	0.16	3.08	0.04	0.09
Oleic (18:1, ω-9), %	1.05	22.9	0.12	0.30
Linoleic (18:2, ω-6), %	2.25	27.1	0.23	0.60
α-Linolenic (18:3, ω-3), %	0.04	2.51	0.08	0.40

^1^Values obtained from a commercial laboratory wet chemistry analysis (Dairy One Forage Laboratory, Ithaca, NY). Total digestible nutrients were calculated according to the equations described by Weiss et al. (1992). Net energy for maintenance was calculated with equations described by the NRC (2000).

^2^Church and Dwight Co., Inc. (Princeton, NJ).

^3^According to Sukhija and Palmquist (1988) using gas chromatography (Autosystem XL Gas Chromatograph, Perkin Elmer, Inc., Waltham, MA).

**Table 2.** Composition and nutritional profile of treatments

Item	CONC	LMB
Ingredients, % dry matter basis		
Cottonseed meal	8.50	8.37
Molasses	60.3	60.4
Essentiom	24.7	24.7
Ca phosphate	3.35	3.36
Mg oxide	3.15	3.17
Nutrient profile, dry matter basis		
Dry matter, %	92.7	89.9
Total digestible nutrients,^2^ %	99	87
Net energy for maintenance,^3^ Mcal/kg	2.46	2.20
Crude protein, %	9.75	9.30
Neutral detergent fiber, %	3.14	3.40
Fatty acids, %	21.0	21.9
Palmitic (16:0), %	6.53	6.57
Stearic (18:0), %	0.80	0.91
Oleic (18:1, ω-9), %	5.81	5.59
Linoleic (18:2, ω-6), %	7.02	7.17
α-Linolenic (18:3, ω-3), %	0.67	0.80

^1^CONC = hand-fed granular supplement enriched with Ca salts of soybean oil (Essentiom, Church and Dwight Co., Inc., Princeton, NJ); LMB = low-moisture molasses-based block enriched with Ca salts of soybean oil (Essentiom, Church and Dwight Co., Inc.). Results are based on individual ingredients of the CONC, and LMB sample collected prior to the beginning of the experiment.

^2^Calculated according to the equations described by Weiss et al. (1992).

^3^Calculated with equations described by the NRC (2000).

### Sampling and Laboratorial Analyses

Samples of hay, LMB, and ingredients from the CONC treatment were collected before the beginning of the experiment and analyzed for nutrient concentration by a commercial laboratory (Dairy One Forage Laboratory, Ithaca, NY). All samples were analyzed by wet chemistry procedures for concentrations of crude protein (method 984.13; AOAC, 2006), acid detergent fiber (method 973.18 modified for use in an Ankom 200 fiber analyzer, Ankom Technology Corp., Fairport, NY; AOAC, 2006), neutral detergent fiber (Van Soest et al., 1991; modified for use in an Ankom 200 fiber analyzer, Ankom Technology Corp.), and FA concentrations using gas chromatography (Autosystem XL Gas Chromatograph, Perkin Elmer, Inc., Waltham, MA) according to Sukhija and Palmquist (1988). Only FA that were individually identified in the analysis are reported. Calculations for total digestible nutrients used the equations proposed by Weiss et al (1992), whereas net energy for maintenance and gain were calculated with the equations proposed by the NRC (2000).

During each experimental period (days 0 to 42), hay DM intake was recorded daily from each pen by collecting and weighing offered and nonconsumed hay (0700 h). All samples were dried for 24 h at 70 °C in forced-air ovens to calculate DM. Pens assigned to CONC received treatments once daily (0730 h) prior to the hay feeding, whereas CONC was consumed by cows within 30 min of feeding. One LMB (90.9 kg, as-fed basis; 58.7 cm diameter × 41.2 cm height) was placed in the back of each drylot back assigned to this treatment, in a manner that cows could access the LMB from all sides. From days 0 to 13 of each period, the LMB was not weighed to allow cows to adapt and consume blocks without interference from research personnel. The LMB was weighed every other day (0730 h) from days 14 to 42 and divided by 2 to represent daily intake. The LMB was replaced by a new one once it reached 10% of its original weight. The CONC was offered at 0.454 kg/cow daily (as-fed basis; 0.420 kg of DM/cow daily) from days 0 to 13 and adjusted (days 14 to 42) in 0.057 kg/cow (as-fed basis) increments/decrements every 2 d to match LMB intake. This adjustment rate was adopted to minimize daily variation in CONC intake, complying with intake behavior typical of hand-fed granular supplements (Bowman and Sowell, 1997).

Cow BW and BCS (Wagner et al., 1988) were recorded, and blood samples were collected on days 0, 14, 28, and 42 of each period. Blood was collected from the coccygeal vein or artery into blood collection tubes (Vacutainer, 10 mL; Becton Dickinson, Franklin Lakes, NJ) containing freeze-dried sodium heparin. Blood samples were placed immediately on ice after collection, centrifuged (2,500 × g for 30 min; 4 °C) for plasma harvest and stored at −80 °C on the same day of collection. Plasma samples were analyzed for FA concentration using gas chromatography (Agilent 7890, Agilent Technologies, Inc.; Santa Clara, CA) using the procedures described by Brandão et al. (2018). Only FA that were individually identified in the analysis are reported.

### Statistical Analysis

All data were analyzed using pen as experimental unit, Satterthwaite approximation to determine the denominator degrees of freedom for tests of fixed effects, and the MIXED procedure of SAS (SAS Inst. Inc., Cary, NC). Model statements contained the effects of treatment, time variable, the treatment × time interaction, in addition to period and block as independent variables. Intake results were analyzed using pen (treatment × period) as random variable, whereas all other results used pen (treatment × period) and cow (pen) as random variables. For analyses using repeated measures, the specified term was day, whereas the subject was pen (treatment × period) for intake results and cow (pen) for all other variables. The covariance structure utilized was autoregressive, which provided the best fit for these analyses according to the lowest Akaike information criterion. All results are reported as least square means, and least square differences or PDIFF were used for simple or multiple mean separation, respectively. Significance was set at *P* ≤ 0.05, and tendencies were determined if *P* > 0.05 and ≤ 0.10.

## RESULTS AND DISCUSSION

**Figure 1.**
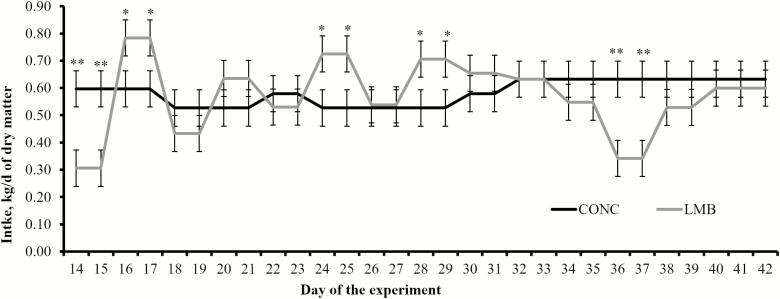
Intake of a molasses-based supplement enriched with Ca salts of soybean oil (24.7% of dry matter; Essentiom, Church and Dwight Co., Inc., Princeton, NJ) and delivered to beef cows via self-fed low-moisture block (LMB; *n* = 6 pens) or via hand-fed granular concentrate daily (CONC; *n* = 6 pens). Supplemented treatments were provided from days 0 to 42. The LMB was not weighed from days 0 to 13 to allow cows to adapt and consume blocks without interference from research personnel. The LMB was weighed every other day from days 14 to 42 and divided by 2 to represent daily intake. The CONC was offered at 0.454 kg/cow daily (as-fed basis; 0.420 kg of DM/cow daily) from days 0 to 13 and adjusted (days 14 to 42) in 0.057 kg/cow (as-fed basis) increments/decrements every 2 d to match LMB intake. A treatment × day interaction was detected (*P* < 0.01). Within days, **P < 0.01 and *P ≤ 0.05.

Supplementing LMB to cattle requires an adaptation period to ensure that animals recognize the LMB as a feed source and learn how to consume the supplement (Garossino et al., 2003; Moriel et al., 2019). For this reason, LMB intake from days 0 to 13 was not measured to prevent external interferences that affect adaptation of cows to LMB. Yet, daily LMB intake during the initial 13 d was 0.846 ± 0.107 kg/cow (DM basis), and double the designed LMB intake and concurrent CONC supplementation rate (0.420 kg/cow daily; DM basis). These outcomes may be associated with the curiosity and competition of cows to explore LMB, given that cows had no previous experience with this supplementation strategy. The LMB can also be perceived as an environmental enrichment by confined cattle, and its intake heightened in cows adapting to drylot conditions (Pelley et al., 1995). Corroborating these outcomes, Moriel et al. (2019) reported greater LMB intake during the first week of supplementation in drylotted beef heifers compared to subsequent weeks. From days 14 to 42 of the experimental period, supplement intake was designed to be similar and indeed did not differ (*P* ≥ 0.21) between LMB and CONC cows (Table 3). The expected variation in daily intake of LMB (Bowman and Sowell, 1997) resulted in a treatment × day interaction (*P* < 0.01) described in Fig. 1. Intake of LMB remained greater than anticipated after day 14, suggesting that cows continued to perceive the supplement as environmental enrichment (Pelley et al., 1995). Alternatively, the LMB intake observed in this experiment may have represented the actual intake of the supplement. The LMB was designed to yield a daily intake of 0.454 kg (as-fed basis; 0.408 kg of DM/cow daily) in grazing cattle, but no grazing cows were evaluated herein to serve as reference for LMB intake.

**Table 3.** Feed intake, body weight, and body condition score of forage-fed beef cows receiving no supplementation (NOSUPP; *n* = 6 pens) or receiving a molasses-based supplement enriched with Ca salts of soybean oil (24.7% of dry matter; Essentiom, Church and Dwight Co., Inc., Princeton, NJ) via self-fed low-moisture block (LMB; *n* = 6 pens) or hand-fed granular concentrate daily (CONC; *n* = 6 pens). Supplement treatments were provided from days 0 to 42 of the experiment^1^

Item	NOSUPP	CONC	LMB	SEM	*P*-value
Supplement intake, kg/d (DM basis)					
Days 14 to 28	—	0.554	0.564	0.017	0.68
Days 28 to 42	—	0.611	0.575	0.018	0.21
Overall (days 14 to 42)	—	0.583	0.570	0.011	0.39
Hay intake, kg/d (DM basis)	13.8	13.6	13.2	0.5	0.59
Body condition score^2^					
Day 0	5.54	5.58	5.54	0.11	0.95
Day 14	5.71	5.79	5.79	0.11	0.83
Day 28	5.89	6.00	5.85	0.11	0.64
Day 42	6.17	6.33	6.14	0.11	0.44
Total gain (days 0 to 42)	0.62	0.75	0.60	0.08	0.40
Body weight, kg					
Day 0	464	463	465	20	0.99
Day 14	478	476	477	20	0.99
Day 28	489	486	496	20	0.93
Day 42	494	482	501	20	0.81
Total gain (days 0 to 42)	29	19	35	11	0.60

^1^Hay intake was recorded daily from each pen by collecting and weighing offered and nonconsumed hay. From days 0 to 13, the LMB was not weighed to allow cows to adapt and consume blocks without interference from research personnel. The LMB was weighed every other day from days 14 to 42, divided by 2 to represent daily intake, and averaged across LMB pens. The CONC was offered at 0.454 kg/cow daily (as-fed basis; 0.420 kg of DM/cow daily) from days 0 to 13 and adjusted (days 14 to 42) every 2 d to match LMB intake.

^2^According to Wagner et al. (1988).

No treatment or treatment × day interactions were noted (*P* ≥ 0.40) for hay intake, BCS, and BW among treatments (Table 3), although CSSO and energy supplements based on molasses-based may depress forage intake and improve BW gain (Brown, 1993; Moore et al., 1999; Cooke et al., 2011). Forage intake, however, is impacted when supplemental TDN intake is >0.70% of BW, sugarcane molasses constitutes >15% of the dietary DM, and supplemental fat is >2% of diet DM (Kalmbacher et al., 1995; Moore et al., 1999; Hess et al., 2008). Based on supplement DM intake from days 0 to 42 of LMB and CONC cows (0.662 and 0.530 kg/cow daily, respectively; SEM = 0.019, *P* < 0.01), supplemental TDN intake was below 0.12% of BW, and sugarcane molasses and supplemental fat represented less than 2.8% and 1.0% of dietary DM, respectively. Based on hay + supplement intake from days 0 to 42 (Table 3), no differences among NOSUPP, LMB, and CONC were noted (*P* ≥ 0.61) for mean daily TDN intake (8.18, 8.32, and 8.19 kg/d, respectively; SEM = 0.28) and daily CP intake (2.43, 2.36, and 2.44 kg/d, respectively; SEM = 0.08). Hence, the supplementation level adopted herein was not sufficient to affect forage intake and provide supplemental energy and protein to change BW and BCS. Nonetheless, this experiment was designed to evaluate LMB as a carrier for CSSO and not to investigate the impacts of LMB and CONC on cattle BW and BCS gain.

Plasma concentrations of FA reflect intake and intestinal FA flow (Klusmeyer and Clark, 1991; Lake et al., 2007; Hess et al., 2008), and FA reach target tissues for accumulation via circulation (Mattos et al., 2000; Wathes et al., 2007; Cooke et al., 2014). For these reasons, the central objective of this study was to compare plasma FA profile of NOSUPP, LMB, and CONC cows throughout the experimental period (Tables 4 to 6). No treatment or treatment × day interactions were detected (*P* ≥ 0.20) for plasma concentrations of myristic acid, palmitoleic acid, oleic acid, arachidonic acid, docosapentaenoic acid, and total monounsaturated FA. Previous research from our group also reported that CSSO supplementation did not increase plasma concentrations of these FA in beef cows (Cooke et al., 2014; Cipriano et al., 2016; Brandão et al., 2018). Treatment × day interactions were detected for all other individual FA and total FA concentrations (*P* ≤ 0.01). Plasma FA profile on day 0 did not differ (*P* ≥ 0.20) between treatments (Tables 4-6), even when periods are analyzed independently (*P* ≥ 0.36; data not shown). Hence, all cows had similar circulating FA profile at the beginning of the experiment, and the washout interval eliminated carryover effects on plasma FA profile from period 1 to 2.

**Table 4.** Plasma concentrations of saturated and monounsaturated fatty acids (µg/mL of plasma) in forage-fed beef cows receiving no supplementation (NOSUPP; *n* = 6 pens), or receiving a molasses-based supplement enriched with Ca salts of soybean oil (24.7% of dry matter; Essentiom, Church and Dwight Co., Inc., Princeton, NJ) via self-fed low-moisture block (LMB; *n* = 6 pens) or hand-fed granular concentrate daily (CONC; *n* = 6 pens). Supplement treatments were provided from days 0 to 42 of the experiment^1^

Item^3^	NOSUPP	CONC	LMB	SEM	*P*-value
Myristic (14:0)	5.29	5.71	4.76	0.45	0.36
Palmitic (16:0)					
Day 0	73.3	69.6	74.2	5.3	0.81
Day 14	80.2^c^	101^b^	119^a^	5.3	<0.01
Day 28	70.4^b^	102^a^	98.4^a^	5.3	<0.01
Day 42	66.1^b^	102^a^	97.4^a^	5.3	<0.01
Palmitoleic (16:1, ω-7)	3.36	3.15	3.08	0.12	0.26
Stearic (18:0)					
Day 0	116	117	114	6	0.91
Day 14	117^c^	138^b^	163^a^	6	<0.01
Day 28	114^b^	152^a^	146^a^	6	<0.01
Day 42	100^b^	137^a^	148^a^	6	<0.01
Oleic (18:1, ω-9)	45.2	49.7	50.3	2.1	0.20

^1^Blood samples were collected on days 0, 14, 28, and 42 for plasma harvest and analyzed for fatty acid concentration according to Brandão et al. (2018).

**Table 5.** Plasma concentrations of polyunsaturated fatty acids (µg/mL of plasma) in forage-fed beef cows receiving no supplementation (NOSUPP; *n* = 6 pens) or receiving a molasses-based supplement enriched with Ca salts of soybean oil (24.7% of dry matter; Essentiom, Church and Dwight Co., Inc., Princeton, NJ) via self-fed low-moisture block (LMB; *n* = 6 pens) or hand-fed granular concentrate daily (CONC; *n* = 6 pens). Supplement treatments were provided from days 0 to 42 of the experiment^1^

Item^3^	NOSUPP	CONC	TUB	SEM	*P*-value
Linoleic (18:2, ω-6)					
Day 0	135	133	128	11	0.88
Day 14	139^c^	245^b^	332^a^	11	<0.01
Day 28	141^b^	306^a^	305^a^	11	<0.01
Day 42	139^b^	313^a^	330^a^	11	<0.01
γ-Linolenic (18:3, ω-6)					
Day 0	5.25	5.02	4.76	0.33	0.58
Day 14	4.41	4.43	3.72	0.33	0.24
Day 28	4.63^b^	6.00^a^	6.09^a^	0.33	<0.01
Day 42	4.24^b^	5.64^a^	6.17^a^	0.33	<0.01
α-Linolenic (18:3, ω-3)					
d 0	67.2	65.9	66.7	3.0	0.95
d 14	65.4^a^	44.6^b^	44.4^b^	3.0	<0.01
d 28	70.0^a^	51.7^b^	51.7^b^	3.0	<0.01
d 42	64.3^a^	46.2^b^	51.7^b^	3.0	<0.01
Dihomo-γ-linolenic acid (20:3, ω-6)					
Day 0	11.3	10.8	10.2	0.8	0.64
Day 14	12.5	13.6	12.4	0.8	0.56
Day 28	11.8^b^	16.3^a^	15.4^a^	0.8	0.02
Day 42	10.5^c^	14.4^b^	17.0^a^	0.8	<0.01
Arachdonic (20:4, ω-6)	19.5	19.8	19.8	0.5	0.93
Docosadienoic (22:2, ω-6)					
Day 0	9.47	9.13	10.0	0.57	0.51
Day 14	10.9^a^	7.97^b^	7.74^b^	0.57	<0.01
Day 28	9.78^a^	7.16^b^	7.03^b^	0.57	<0.01
Day 42	9.67^a^	6.77^b^	6.90^b^	0.57	<0.01
Docosapentaenoic (22:5, ω-3)	9.88	9.41	9.70	0.41	0.71
Osbond (22:5, ω-6)					
Day 0	17.2	16.3	16.1	1.1	0.74
Day 14	17.0^c^	21.1^b^	26.3^a^	1.1	<0.01
Day 28	19.1^b^	26.2^a^	25.9^a^	1.1	<0.01
Day 42	18.7^b^	26.4^b^	29.7^a^	1.1	<0.01

^1^Blood samples were collected on d 0, 14, 28, and 42 for plasma harvest, and analyzed for fatty acid concentration according to Brandão et al. (2018).

**Table 6.** Plasma fatty acid (FA) profile (µg/mL of plasma) in forage-fed beef cows receiving no supplementation (NOSUPP; *n* = 6 pens) or receiving a molasses-based supplement enriched with Ca salts of soybean oil (24.7% of dry matter; Essentiom, Church and Dwight Co., Inc., Princeton, NJ) via self-fed low-moisture block (LMB; *n* = 6 pens) or hand-fed granular concentrate daily (CONC; *n* = 6 pens). Supplement treatments were provided from days 0 to 42 of the experiment^1^

Item^3^	NOSUPP	CONC	TUB	SEM	*P*-value
Total saturated FA					
Day 0	216	218	215	12	0.98
Day 14	228^c^	268^b^	308^a^	12	<0.01
Day 28	215^b^	283^a^	267^a^	12	<0.01
Day 42	191^b^	264^a^	270^a^	12	<0.01
Total monounsaturated FA	52.0	54.9	55.4	2.5	0.60
Total polyunsaturated FA					
Day 0	274	267	264	14	0.87
Day 14	284^b^	368^b^	458^a^	14	<0.01
Day 28	284^b^	444^a^	440^a^	14	<0.01
Day 42	274^b^	448^a^	474^a^	14	<0.01
Total ω-3 polyunsaturated FA					
Day 0	75.9	74.4	76.3	3.2	0.90
Day 14	78.4^b^	55.6^a^	56.5^a^	3.2	<0.01
Day 28	79.5^b^	62.3^a^	61.2^a^	3.2	<0.01
Day 42	73.7^b^	59.0^a^	63.7^a^	3.2	<0.01
Total ω-6 polyunsaturated FA					
Day 0	198	192	188	13	0.84
Day 14	205^c^	312^b^	402^a^	13	< 0.01
Day 28	204^b^	382^a^	379^a^	13	< 0.01
Day 42	201^b^	396^a^	420^a^	13	< 0.01
Total identified FA					
Day 0	542^b^	539^a^	536^a^	27	0.98
Day 14	568^b^	691^a^	827^a^	27	<0.01
Day 28	550^b^	783^a^	761^a^	27	<0.01
Day 42	515^b^	755^a^	797^a^	27	<0.01

^1^Blood samples were collected on days 0, 14, 28, and 42 for plasma harvest and analyzed for fatty acid concentration according to Brandão et al. (2018).

Plasma FA concentrations on days 14, 28, and 42 corroborate the FA content and intake of treatments during the experiment (Tables 4-6). On day 14, plasma concentrations of palmitic acid, stearic acid, linoleic acid, osbond acid, total saturated FA, total polyunsaturated FA, total ω-6 polyunsaturated FA, and total FA were greater (*P* < 0.01) in CONC and LMB vs. NOSUPP cows, and also greater (*P* ≤ 0.03) in LMB vs. CONC cows. Plasma concentrations of α-linolenic acid and total ω-3 polyunsaturated FA on day 14 were greater (*P* < 0.01) in NOSUPP vs. LMB and CONC cows, and did not differ (*P* ≥ 0.84) between the latter two treatments. As previously noted, LMB intake during the initial 14 d were beyond the expected and nearly double the supplement intake of CONC cows, explaining differences observed between these treatments in samples collected on day 14. The decrease in plasma α-linolenic acid and ɷ-3 polyunsaturated FA concentrations in CSSO-supplemented cattle has also been reported by our group in research with mature and growing beef cattle (Cooke et al., 2011; Brandão et al., 2018; Schubach et al., 2019). On days 28 and 42, when CONC intake was adjusted to match LMB intake (Table 3), plasma concentrations of palmitic acid, stearic acid, linoleic acid, γ-linolenic acid, dihomo-γ-linolenic acid, osbond acid, total saturated FA, total polyunsaturated FA, total ω-6 polyunsaturated FA, and total FA were greater in CONC and LMB vs. NOSUPP cows and did not differ (*P* ≥ 0.35) between LMB and CONC cows. Plasma concentrations of α-linolenic acid and ɷ-3 polyunsaturated FA remained greater (*P* < 0.01) in NOSUPP vs. CONC and LMB, and similar (*P* ≥ 0.55) between CONC and LMB. Therefore, cows receiving LMB or CONC had a similar plasma FA profile when receiving the same supplementation rate, and a similar increase in linoleic and its ω-6 polyunsaturated FA derivatives compared with NOSUPP cohorts.

Collectively, inclusion of CSSO into LMB resulted in similar incorporation of ω-6 polyunsaturated and total FA in the circulation compared with CONC consumed at the same rate. These results suggest that the manufacturing process of LMB did not impair the integrity and ruminal stability of CSSO, and the daily variation noted in LMB intake did not influence circulating levels of ω-6 polyunsaturated and total FA (Cook et al., 2017). Therefore, the use of self-fed LMB appears to be a valid strategy to provide CSSO to beef cattle with reduced labor needs. Research is still warranted to evaluate and refine LMB and subsequent CSSO intake by grazing cattle, and determine if providing a CSSO-enriched LMB will improve reproductive and productive responses in cow–calf systems.
